# Thalamocortical Mechanisms Underlying Real and Imagined Acupuncture

**DOI:** 10.3390/biomedicines11071830

**Published:** 2023-06-26

**Authors:** Qiao Kong, Valeria Sacca, Kathryn Walker, Sierra Hodges, Jian Kong

**Affiliations:** Department of Psychiatry, Massachusetts General Hospital, Harvard Medical School, Boston, MA 02129, USA

**Keywords:** acupuncture, pain, functional connectivity, thalamocortical, video-guided acupuncture imagery treatment, e-health

## Abstract

Both acupuncture and imagery have shown potential for chronic pain management. However, the mechanisms underlying their analgesic effects remain unclear. This study aims to explore the thalamocortical mechanisms underlying acupuncture and video-guided acupuncture imagery treatment (VGAIT), a combination of acupuncture and guided imagery, using the resting-state functional connectivity (rsFC) of three thalamic subdivisions—the ventral posterolateral thalamus (VPL), mediodorsal thalamus (MD), and motor thalamus subregion (Mthal)—associated with somatosensory, limbic, and motor circuity. Twenty-seven healthy individuals participated in a within-subject randomized crossover design study. Results showed that compared to sham acupuncture, real acupuncture altered the rsFC between the thalamus and default mode network (DMN) (i.e., mPFC, PCC, and precuneus), as well as the prefrontal and somatosensory cortex (SI/SII). Compared to the VGAIT control, VGAIT demonstrated greater rsFC between the thalamus and key nodes within the interoceptive network (i.e., anterior insula, ACC, PFC, and SI/SII), as well as the motor and sensory cortices (i.e., M1, SMA, and temporal/occipital cortices). Furthermore, compared to real acupuncture, VGAIT demonstrated increased rsFC between the thalamus (VPL/MD/Mthal) and task-positive network (TPN). Further correlations between differences in rsFC and changes in the heat or pressure pain threshold were also observed. These findings suggest that both acupuncture- and VGAIT-induced analgesia are associated with thalamocortical networks. Elucidating the underlying mechanism of VGAIT and acupuncture may facilitate their development, particularly VGAIT, which may be used as a potential remote-delivered pain management approach.

## 1. Introduction

Pain can significantly impact physical and psychological well-being, and is the primary reason for seeking medical intervention. While acute pain may serve as a crucial protective mechanism, alerting us to potential harm or injury, its persistence can give rise to the debilitating state of chronic pain [1]. Acute pain, typically arising from tissue damage or inflammation, manifests swiftly and subsides as the underlying cause heals [2]. In contrast, chronic pain extends beyond the normal healing process and has emerged as a significant public health issue that is notoriously difficult to manage [3].

Given the increased necessity for virtual care visits, the COVID-19 pandemic has further exacerbated the challenges of managing pain [4]. Opioids can be essential medications for pain relief; however, the small to moderate short-term benefits and high risk of adverse effects from opioid use have led both patients with acute or chronic pain and clinicians to seek alternative treatment approaches [5,6].

Recently, acupuncture has gained acceptance as a potential treatment option for acute and chronic pain. For instance, the 2022 CDC clinical practice guidelines recommend nonopioid therapies including acupuncture to help manage subacute and chronic pain [7]. The American College of Physicians (ACP) and the American Academy of Family Physicians (AAFP) also recommend acupressure to improve pain and function [8]. While acupuncture has been shown to provide therapeutic analgesia, access to treatment can be limited by cost and the inconvenience of in-person visits.

Combining acupuncture and imagery, we have developed a new method for care delivery called video-guided acupuncture imagery treatment (VGAIT) [9]. Previous studies have suggested that VGAIT may provide pain relief in both healthy and chronic low back pain participants [10,11]. However, the underlying neural mechanism of VGAIT remains unclear.

The thalamocortical (TC) circuit has been found to play a key role in the pathophysiology of chronic pain. Previous studies have revealed that individuals with chronic pain experience altered thalamocortical rhythm, known as thalamocortical dysrhythmia (TCD) [12], as well as abnormal connectivity within the TC network [13,14]. Several lines of evidence have suggested that some treatments may achieve analgesic effects by modulating TC circuits. For instance, non-invasive neuromodulation techniques, such as transcranial alternating current stimulation (tACS) and transcutaneous vagus nerve stimulation (taVNS), have been shown to modulate TCD while inducing pain relief to treat chronic low back pain and migraine [15,16].

The literature on acupuncture mechanisms suggests that treatment-related analgesia may be achieved through the complex loops between thalamic nuclei and cortical regions, making it an important target for investigation [17,18]. Furthermore, according to research, mental imagery can be a potent modulator of thalamic activity [19]. For example, a previous neuroimaging study demonstrated that the strongest activation signature associated with mental imagery was in the thalamus [20]. Despite the emerging evidence of these correlations, the analgesic effects underlying acupuncture and VGAIT in terms of TC functional interactions are not yet well understood. 

The thalamus structure is not uniform and consists of several nuclei with highly specific functions and connectivity to distinct cortical regions [21,22,23]. The lateral thalamus, mainly the ventral posterolateral thalamus (VPL), is the principal sensory nucleus of the thalamus and sends projections to the somatosensory cortices, while the mediodorsal thalamus (MD), which is associated with the emotional and affective components of pain, plays a vital role in the limbic circuity and connects primarily to limbic cortices [24]. In addition, motor control dysfunction is common across many chronic pain conditions. The primary motor cortex (M1) is the most commonly used target with neuromodulation techniques for chronic pain treatment, which demonstrates the role of the motor system in pain management [25,26]. The motor thalamus subregion (Mthal), composed of the ventral anterior (VA) and ventral lateral (VL) thalamic nuclei, is a central node bridging subcortical and cortical motor circuits and plays an important role in the regulation of pain-related motor control [27,28]. Furthermore, evidence suggests that somatosensory input can evoke motor cortical responses [29]. Thus, as a form of somatosensory stimulation, acupuncture may modulate corticomotor excitability [30]. 

Based on previous studies, we hypothesized that both acupuncture and VGAIT could modulate the functional connectivity of three thalamic subdivisions associated with somatosensory, limbic circuity, and motor cortex to produce analgesia via distinct neural pathways. To test this hypothesis, we performed seed-based resting-state functional connectivity (rsFC) analysis to compare the different TC patterns across four interventions (real acupuncture, sham acupuncture, VGAIT, and VGAIT control) from twenty-four healthy participants. Then, we explored the association between the rsFC changes and the corresponding changes in pain threshold.

## 2. Material and Methods

Full details of the experimental design and procedures have been reported in our previous study, in which we investigated the fMRI signal changes modulated by real and imagined acupuncture (VGAIT) [10] and their modulation effects on brain regional connectivity [31] and functional connectivity of periaqueductal gray (PAG) and ventral tegmental area (VTA) [32]. This study aims to extend this line of research by investigating how acupuncture and VGAIT can modulate thalamocortical circuits, which has never been published before. 

### 2.1. Participants

Twenty-seven healthy, right-handed, and acupuncture-naive individuals (age range: 19–33 years; 18 females) were recruited in the cross-over study. This study was approved by the Partners Human Research Committee (IRB) of Massachusetts General Hospital (MGH). All participants signed the consent form prior to the start of the study.

### 2.2. Experiment Procedures

Participants went through five experimental sessions. Session 1 was a training session. Sessions 2–5 were the intervention sessions, during which the participants received four interventions in a random order: (a) real acupuncture, (b) sham acupuncture, (c) VGAIT, and (d) VGAIT control. The intervals between each intervention were at least seven days. 

#### 2.2.1. Session 1 Training Session

All participants were trained in Quantitative Sensory Testing (QST) to assess heat and pressure pain thresholds. They then received real acupuncture needles at right acupoints SP6 (Sanyinjiao) and SP9 (Yinlingquan), as well as cotton swab interventions at non-acupoints. The interventions were videotaped as VGAIT and VGAIT control treatments separately.

#### 2.2.2. Sessions 2–5 Intervention Sessions

Real acupuncture was applied at the right acupoints SP6 and SP9. Sham acupuncture was applied at two sham points (next to SP6/SP9) using a specially designed needle that did not penetrate the skin (Streitberger needle) [33,34]. For each participant, acupoint location, and needling parameters were kept consistent. The total treatment time lasted about 20 min.

In the VGAIT and VGAIT control sessions, participants were given instructions for the imagery acupuncture treatment before watching videotapes recorded in session 1. The intervention time for VGAIT and VGAIT control was identical to real and sham acupuncture (see detailed descriptions of the interventions in Supplementary Material).

### 2.3. Pain Threshold Measurements

Heat and pressure pain thresholds were measured on two different locations (heat pain: right leg and left arm; pressure pain: right leg and left thumbnail) before and after each intervention. We chose both local and distal pain thresholds because they may represent segmental analgesic effects and suprasegmental analgesic effects, respectively [35].

This measurement was repeated three times on each site, with the thermode (heat) and algometer (pressure) repositioned between each threshold assessment. Heat-evoked pain thresholds were assessed using a PATHWAY CHEPS (Contact Heat-Evoked Potential Stimulator, Medoc Advanced Medical Systems) [36] and pressure-evoked pain thresholds were assessed using an algometer [37]. The mean values of the three assessments at each site were calculated and used as the final pain threshold value (see Supplementary Material and previous publication [38] for the details of the QST procedure).

### 2.4. MRI Data Acquisition

All MRI data were collected with a 32-channel head coil and 3T scanner (Siemens, Skyra syngo) at the Martinos Center for Biomedical Imaging. This study focused on resting-state MRI scans before and after each intervention. Resting-state functional MRI (fMRI) data were obtained with an echo-planar imaging sequence under the following acquisition parameters: repetition time (TR) = 3000 ms, echo time (TE) = 30 ms, flip angle = 90°, slice thickness = 3 mm, slice numbers: 44, voxel size = 3 × 3 × 3 mm^3^, field of view: 192 × 192 mm^2^, total 164 volumes. High-resolution T1-weighted images were acquired with the magnetization-prepared rapid gradient echo (MPRAGE) sequence with the following parameters: TR = 2530 ms, TE = 1.69 ms, flip angle = 7°, slice thickness = 1 mm, slice numbers: 176, voxel size = 1 × 1 × 1 mm^3^, field of view: 256 × 256 mm^2^. The participants were instructed to keep their eyes open, blink normally, and remain still during the fMRI scan.

### 2.5. MRI Data Pre-Processing

Resting-state fMRI data were preprocessed using the CONN toolbox version 21a (http://www.nitrc.org/projects/conn, accessed on 24 January 2023). The preprocessing pipeline was as follows: removal of the first five volumes, slice-timing correction, realignment, outlier detection, indirect segmentation and normalization (MNI 152 template), smoothing with a Gaussian kernel of 6 mm full-width half-maximum (FWHM), regression of nuisance covariates and head motion scrubbing, linear detrending, and filtering with a band-pass frequency window of 0.008–0.09 Hz.

### 2.6. Seed-Based Functional Connectivity Analysis

Three thalamic subdivisions were included as seeds based on the parcellation of the thalamic nuclei of AAL3 [39]: seed 1: sensory thalamus (VPL); seed 2: limbic thalamus (MD); seed 3: motor thalamus (VA, VL).

Functional connectivity analysis was computed between each seed and every other voxel in the brain. In the first-level analysis, correlation maps were produced for each subject by extracting the time course of the BOLD signal from each seed and by computing Pearson’s correlation coefficients between the time courses in the seeds and all other brain voxels. Correlation coefficients were transformed into z-scores to increase normality. In the group-level analysis, paired *t*-tests were used to compare functional connectivity between different time points and interventions, respectively (e.g., post vs. pre; VGAIT vs. VGAIT control). A voxel-level threshold at *p* < 0.005 and a cluster-level False Discovery Rate (FDR) of *p* < 0.05 were applied. 

To explore the association between the pain threshold changes and corresponding functional connectivity changes, we extracted the rsFC z-scores from brain regions with significantly altered rsFC based on different contrasts (i.e., VGAIT post vs. pre; Real acupuncture post vs. pre; Real vs. sham acupuncture post minus pre; VGAIT vs. VGAIT control post minus pre). The correlation between these rsFC values (z-score) and pain threshold changes was estimated by Pearson’s correlation (*r*). Statistical significance was set at *p* < 0.05, and Bonferroni correction was applied for accounting for the multiple comparisons. Statistical analysis was performed using JASP version 0.17.1 (http://www.jasp-stats.org, accessed on 24 January 2023).

## 3. Results

Twenty-four participants (mean ± standard deviation, 25.21 ± 3.83 years of age; 16 females) out of the twenty-seven initially enrolled participants completed the study and were included in the analysis.

### 3.1. Seed-Based rsFC Results

#### 3.1.1. Sensory Thalamus (VPL)

**Figure 1:**
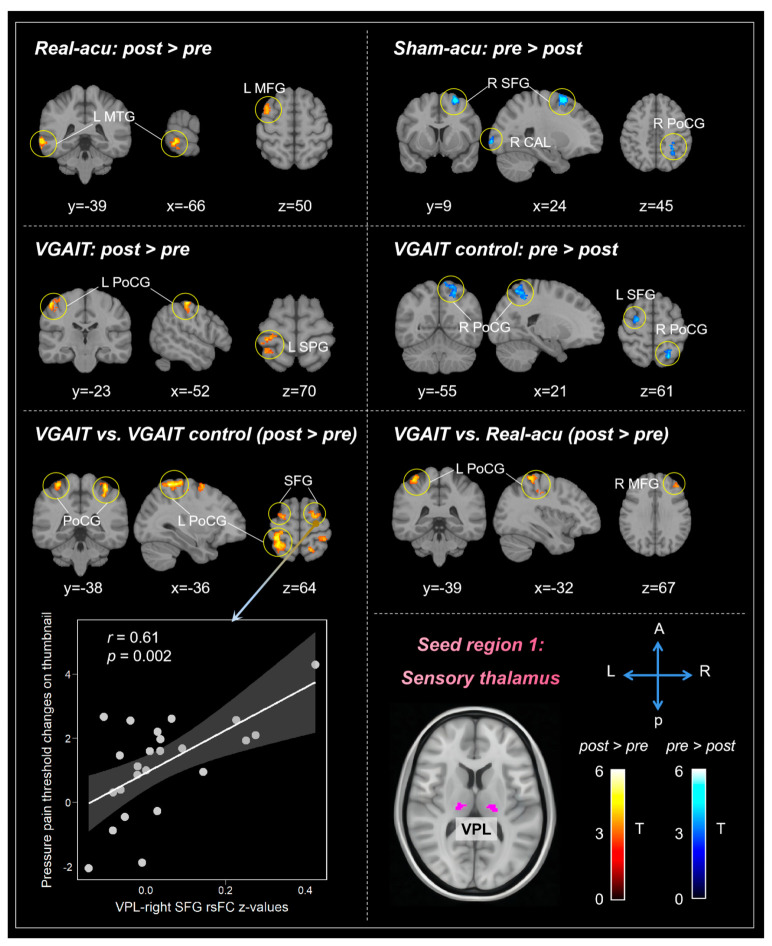
VPL-based rsFC results across different interventions. Pearson correlation scatterplot between VPL-right SFG rsFC change and the corresponding pressure pain threshold changes on the thumbnail (bottom left). Color bar indicates the t value of the comparisons. Abbreviations: L: left, R: right, A: anterior, P: posterior, VPL: ventral posterolateral thalamus, MTG: middle temporal gyrus, MFG: middle frontal gyrus, SFG: superior frontal gyrus, CAL: calcarine, PoCG: postcentral gyrus, SPG: superior parietal gyrus.

We first compared VPL-based rsFC changes before and after each intervention. Results showed that real acupuncture was associated with increased rsFC between the VPL and left middle temporal gyrus (MTG) and middle frontal gyrus (MFG); sham acupuncture was associated with decreased rsFC between the VPL and right superior frontal gyrus (SFG), Calcarine (CAL), and postcentral gyrus (PoCG); VGAIT showed increased rsFC between the VPL and left PoCG and superior parietal gyrus (SPG); and VGAIT control showed decreased rsFC between the VPL and right PoCG and left SFG (Table 1, Figure 1).

We also compared rsFC pre- and post-differences between real and sham acupuncture, VGAIT and VGAIT control, as well as VGAIT and real acupuncture. The results showed that compared to VGAIT control, VGAIT showed increased rsFC between the VPL and bilateral PoCG and dorsolateral prefrontal cortex (DLPFC). Compared to real acupuncture, VGAIT showed increased rsFC between the VPL and left PoCG and right MFG. No significant rsFC changes between the real and sham acupuncture group were observed at the threshold we set (Table 1, Figure 1).

#### 3.1.2. Limbic Thalamus (MD)

Comparisons between post- and pre-treatment in each intervention showed that real acupuncture produced increased rsFC between the MD and several brain regions, including the bilateral middle temporal gyrus (MTG), angular gyrus (ANG), right MFG, inferior frontal gyrus (IFG), cerebellar Lobule VI (CER6), lingual gyrus (LING), left middle occipital gyrus (MOG); and decreased rsFC between the MD and the left precuneus. Sham acupuncture produced decreased rsFC between the MD and right posterior cingulate cortex (PCC), bilateral cerebellum lobule IX (CER9), and MTG. 

**Figure 2:**
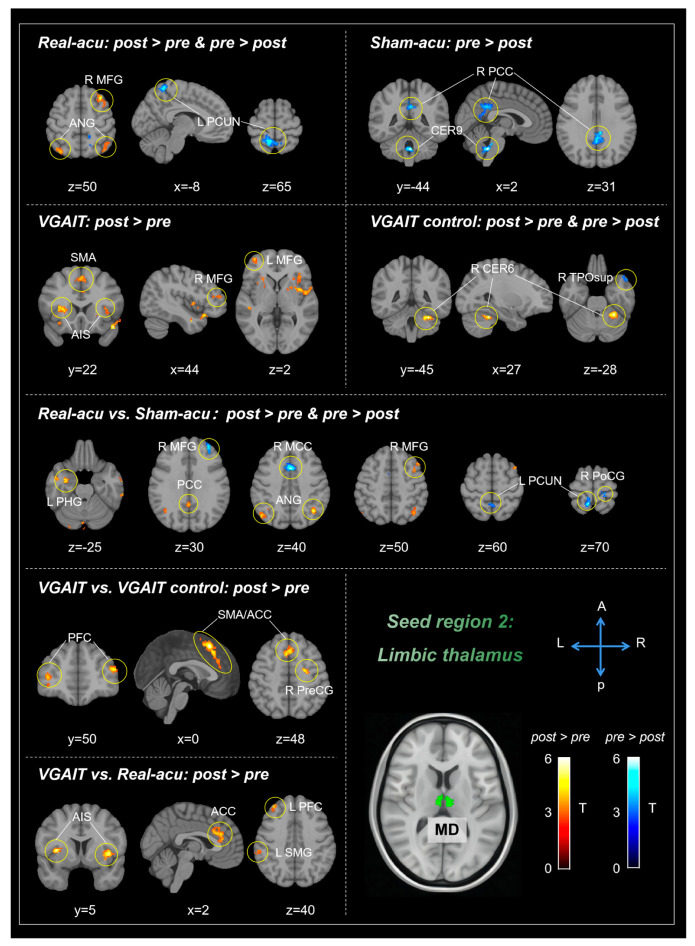
MD-based rsFC results across different interventions. Abbreviations: L: left, R: right, A: anterior, P: posterior, MD: mediodorsal thalamus, MFG: middle frontal gyrus, ANG: angular gyrus, PCUN: precuneus, PCC: posterior cingulate cortex, CER9: Cerebellum lobule IX, SMA: supplementary motor area, AIS: anterior insula, CER6: cerebellum lobule IX, TPOsup: superior temporal pole, PHG: parahippocampal gyrus, MCC: middle cingulate cortex, PFC: prefrontal cortex, ACC: anterior cingulate cortex, SMG: supramarginal gyrus.

VGAIT yielded increased rsFC between the MD and the bilateral anterior insula (AIS), supplementary motor area (SMA), MFG, right superior temporal pole (TPOsup), and left superior temporal gyrus (STG). VGAIT control yielded increased rsFC between the MD and the right CER6, as well as decreased rsFC between the MD and the right TPOsup (Table 2, Figure 2).

Comparisons between the different interventions before and after intervention indicated that real acupuncture showed increased rsFC between the MD and bilateral LING, ANG, PCC, right MTG, MFG, and left parahippocampal gyrus (PHG), as well as decreased rsFC with the left precuneus, right middle cingulate cortex (MCC), MFG, and PoCG, compared to sham acupuncture. VGAIT showed increased rsFC between the MD and the right TPOsup, bilateral SMA/ACC, prefrontal cortex (PFC), and right precentral gyrus (PreCG) compared to the VGAIT control group. Furthermore, VGAIT showed increased rsFC between the MD and the bilateral AIS, ACC, left supramarginal gyrus (SMG), and PFC compared to real acupuncture (Table 2, Figure 2).

#### 3.1.3. Motor Thalamus (Mthal)

**Figure 3:**
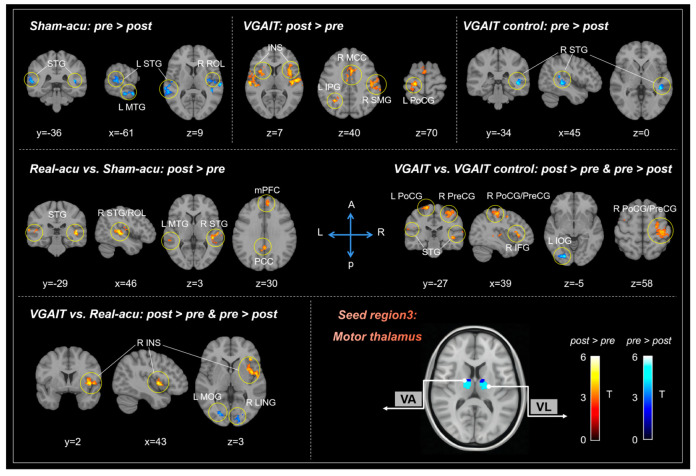
Mthal-based rsFC results across different interventions. Abbreviations: L: left, R: right, A: anterior, P: posterior, Mthal: motor thalamus, STG: superior temporal gyrus, MTG: middle temporal gyrus, ROL: Rolandic operculum, INS: insula, IPG: inferior parietal gyrus, MCC: middle cingulate cortex, SMG: supramarginal gyrus, PoCG: postcentral gyrus, PreCG: precentral gyrus, PCC: posterior cingulate cortex, mPFC: medial prefrontal cortex, IOG: inferior occipital gyrus, IFG: inferior frontal gyrus, MOG: middle occipital gyrus, LING: lingual gyrus.

When comparing Mthal-based rsFC changes before and after each treatment, we found that sham acupuncture produced decreased rsFC between the Mthal and the bilateral STG, MTG, and right Rolandic operculum (ROL). VGAIT yielded increased rsFC between the Mthal and bilateral insula, right MCC, left STG, right SMG, left PoCG, and inferior parietal gyrus (IPG). The VGAIT control produced reduced rsFC between the Mthal and right STG. No significant rsFC changes were in the real acupuncture group before and after the intervention at the threshold we set (Table 3, Figure 3).

We also found that real acupuncture demonstrated increased rsFC between the Mthal and the bilateral STG, PCC, left MTG, right ROL, and medial prefrontal cortex (mPFC) compared to sham acupuncture. VGAIT demonstrated increased rsFC between the Mthal and the bilateral PoCG, PreCG, STG, MTG, right inferior temporal gyrus (ITG), and IFG, as well as reduced rsFC between the Mthal and left inferior occipital gyrus (IOG), compared to the VGAIT control group. Furthermore, VGAIT showed enhanced rsFC between the Mthal and the right insula, as well as reduced rsFC between the Mthal and the right LING and left MOG, when compared to real acupuncture (Table 3, Figure 3).

### 3.2. Pain Threshold Results

**Figure 4:**
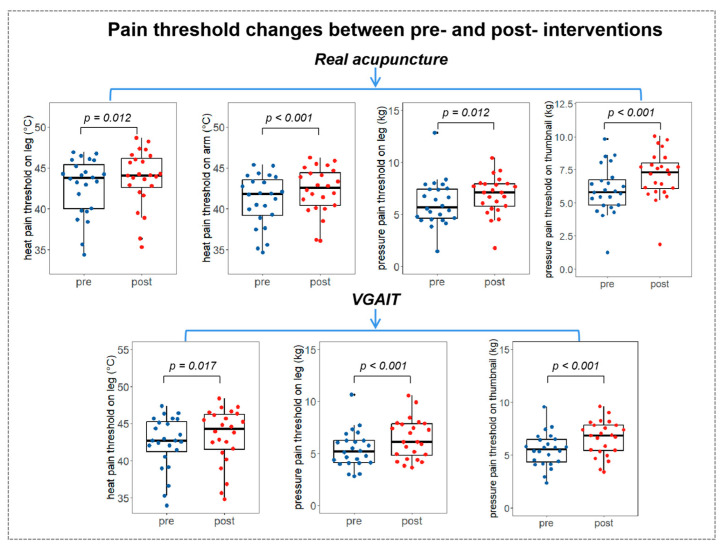
Pain threshold changes between pre- and post-interventions. **Notes**: Pairwise *t*-tests were performed. The boxplot shows the mean ± SD of pain thresholds. Abbreviations: VGAIT, video-guided acupuncture imagery treatment.

Paired samples *t*-test (Student’s *t*-test) on pre- and post-pain threshold differences across four interventions showed that real acupuncture significantly increased the pain threshold for all four measurements (*p* < 0.05). VGAIT significantly increased the heat pain threshold on the leg (*p* = 0.017), and the pressure pain threshold on the leg (*p* < 0.001) and the thumbnail (*p* < 0.001). There were no significant results in sham acupuncture and VGAIT control conditions (see Figure 4 and previous publication [10] for the details of pain threshold changes after different interventions).

**Figure 5:**
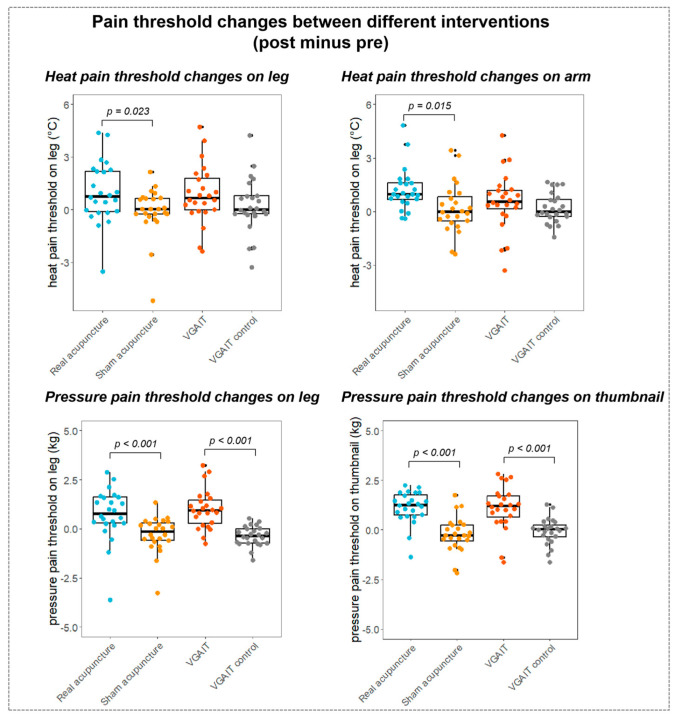
Pain threshold changes between different interventions (post minus pre). **Notes**: pairwise *t*-tests were performed between real and sham acupuncture (post minus pre), and VGAIT and VGAIT control (post minus pre); no significant results were found between VGAIT and real acupuncture. The boxplot shows the mean ± SD of pain thresholds.

Student’s *t*-test on pain threshold changes (post minus pre) between different conditions/interventions revealed that (1) compared to sham acupuncture, real acupuncture significantly increased the pain threshold for all four measurements (heat pain on the leg, *p* = 0.023; heat pain on the arm, *p* = 0.015, pressure pain on the leg, *p* < 0.001, pressure pain on the thumbnail, *p* < 0.001); (2) compared to the VGAIT control, VGAIT significantly increased the pressure pain threshold (pressure pain on the leg, *p* < 0.001, pressure pain on the thumbnail, *p* < 0.001) but not the heat pain threshold (heat pain on the leg, *p* = 0.09; heat pain on the arm, *p* = 0.35); (3) no significant results were observed between VGAIT and real acupuncture (*p* > 0.05) (Figure 5).

### 3.3. Associations between rsFC Differences and Pain Threshold Changes

We further explored the associations between the significant differences in rsFC and the corresponding changes in pain thresholds derived from within-subjects contrasts, including real acupuncture and VGAIT (post vs. pre), real acupuncture vs. sham acupuncture, and VGAIT vs. VGAIT control (post minus pre). 

#### 3.3.1. Correlation between VPL-Based rsFC and Pain Threshold Changes

For VGAIT vs. VGAIT control, the results showed: (1) a significant positive correlation between VPL-left PoCG rsFC and pressure pain threshold changes on the leg (*r* = 0.44, *p* = 0.03, uncorrected); (2) a significant positive correlation between VPL-left SFG rsFC and pressure pain threshold changes on the thumbnail (*r* = 0.48, *p* = 0.02, uncorrected); and (3) a significant positive correlation between VPL-right SFG rsFC and pressure pain threshold changes on the thumbnail (*r* = 0.61, *p* = 0.002, significant after Bonferroni’s correction, *p* < 0.05/10 = 0.005) (Figure 1). No significant correlation between VPL-based rsFC and pain threshold changes was found for real acupuncture and VGAIT (post vs. pre).

#### 3.3.2. Correlation between MD-Based rsFC and Pain Threshold Changes

For real acupuncture (post vs. pre), a significant negative correlation was observed between MD-left precuneus rsFC and heat pain threshold changes on the leg (*r* = −0.48, *p* = 0.02, uncorrected). No significant correlation between MD-based rsFC and pain threshold changes was found in VGAIT (post vs. pre).

For real vs. sham acupuncture, a significant negative correlation between MD-right MFG rsFC and heat pain threshold changes on the arm was observed (*r* = −0.42, *p* = 0.04, uncorrected). There was no significant correlation between MD-based rsFC and pain threshold changes for VGAIT vs. VGAIT control (post minus pre).

#### 3.3.3. Correlation between Mthal-Based rsFC and Pain Threshold Changes

For real vs. sham acupuncture, a significant negative correlation was observed between Mthal-right ROL rsFC and heat pain threshold changes on the arm (*r* = −0.44, *p* = 0.03, uncorrected). For VGAIT vs. the VGAIT control, a significant positive correlation was observed between the Mthal-right IFG rsFC and pressure pain threshold changes on the thumbnail (*r* = 0.42, *p* = 0.04, uncorrected). No significant correlation was observed between Mthal-based rsFC and pain threshold changes for VGAIT (post vs. pre).

## 4. Discussion

In this study, we examined the modulation effects of acupuncture and VGAIT on thalamocortical circuits via functional connectivity changes in three thalamic nuclei. We found that compared to sham acupuncture, real acupuncture exhibited altered rsFC between the thalamus and key regions in the DMN (i.e., mPFC, PCC, precuneus, ANG, and PHG), as well as the prefrontal and somatosensory cortices. Compared to the VGAIT control, VGAIT can significantly alter rsFC between the thalamus and brain regions involved in interoceptive processing, including the anterior insula, sensorimotor, and prefrontal cortex, as well as other sensory cortices. Compared to real acupuncture, VGAIT increased rsFC between the thalamus and the anterior insula, ACC, somatosensory, and prefrontal cortex. Our results suggest that both real acupuncture and VGAIT can modulate the thalamocortical circuits, but each with distinct pathways. 

### 4.1. The Modulation Effects of Acupuncture on Thalamocortical Circuits 

We found that real acupuncture can significantly modulate the rsFC of the thalamus with brain regions within the DMN, compared to sham acupuncture. Specifically, there was an increase in rsFC between the MD and ANG/PCC/PHG, and between the Mthal and mPFC/PCC/lateral temporal cortex, as well as a decrease in rsFC between the MD and precuneus/MCC.

Accumulating evidence indicates that the DMN can be modulated by acupuncture in various diseases, including pain [40]. Recently, researchers optimized a neuroanatomical model of the DMN that identified the anterior thalamus and the MD as its major subcortical nodes [41]. A 7T fMRI study indicated that MD modulation of the DMN cortical activation occurs specifically during internally focused cognition [42]. The mPFC and PCC/precuneus are midline cores of the DMN, which have been shown to play an important role in maintaining pain inhibition efficiency in both healthy and chronic pain conditions [43]. DMN-associated subcortical regions, such as the anterior portion of the thalamus (VA and VL nucleus), are altered in anatomical and functional connectivity in many chronic pain disorders, including chronic low back pain, migraine, trigeminal neuralgia, and others [13,44,45]. In fact, increased thalamic connectivity to the precuneus/PCC has been found in fibromyalgia patients, which is correlated with greater clinical pain changes [46]. In addition, a recent study revealed that mindfulness meditation-induced analgesia was moderated by greater thalamus/precuneus decoupling [47], further suggesting that regulating the connectivity of these regions is crucial for pain modulation.

Additionally, we found that real acupuncture reduced rsFC between the MD and prefrontal and somatosensory cortex, and enhanced rsFC between the Mthal and parietal operculum. Increased MD input to the prefrontal and somatosensory cortex may contribute to chronic pain due to the constant perception of pain [48]; regulating this activity through acupuncture treatment therefore presents a unique opportunity to affect chronic pain states. In addition, the parietal operculum has been recognized as a multisensory integration area that is involved in emotional processing in pain conditions such as fibromyalgia [49,50]. One fMRI study proposed that the parietal operculum serves as an important relay station related to the affective-motivational aspects of pain [51]. Involvement of the parietal operculum may also be associated with the perception and cognitive evaluation of pain [52]. The enhanced rsFC between this region and the Mthal in our study suggests the potential for acupuncture to impact this affective/cognitive-motivational pain pathway. 

In summary, our findings suggest that acupuncture can significantly modulate thalamocortical circuits that may potentially underlie its analgesic effects. 

### 4.2. The Modulation Effects of VGAIT on Thalamocortical Circuits 

We found that VGAIT exhibited enhanced rsFC between the thalamus (VPL/MD/Mthal) and PFC/ACC/somatosensory cortex compared to the VGAIT control; the changes in VPL-SFG rsFC were positively correlated with pressure pain relief. Moreover, VGAIT exhibited a significantly increased rsFC of the Mthal-AIS after treatment. 

The thalamus, a key subcortical relay structure, projects to high-order cortical regions, such as the bilateral primary and secondary somatosensory cortices (SI/SII), prefrontal cortex, ACC, and insula. Interestingly, many of these regions are critical nodes in the interoceptive network [53], suggesting VGAIT may modulate brain pathways associated with interoception.

Interoception is referred to as the process by which the nervous system senses, integrates, interprets, and regulates information about the inner state of the body [54]. It is implicated in a broad range of internal functions, including pain regulation [55]. Altered functional connectivity within the interoceptive neural network has been observed in individuals with migraine, fibromyalgia, primary dysmenorrhea, and other chronic pain conditions [56,57,58,59]. 

The literature suggests mind–body interventions may achieve their treatment effect by regulating these interoceptive neural circuits [60]. For example, a previous study showed that mindfulness training-induced pain relief was associated with the conscious modulation of interoception via higher-order neural networks (i.e., somatosensory cortex, insula, and ACC) [61]. A systematic review of cognitive and meditative therapies (CMT), including cognitive behavioral therapy, mindfulness, and meditation, revealed increased activation of brain regions involved in interoception (i.e., insula and somatosensory cortex) in a chronic pain population following CMT [62]. In line with these findings, our results suggest that VGAIT, which involves viewing and imagining oneself receiving acupuncture, could modulate the interoceptive system to relieve pain.

Furthermore, the ACC is also a key component of the limbic system: it receives afferent inputs primarily from the medial thalamic nuclei, exhibits robust connections with the amygdala, hippocampus, and anterior insula; and is involved in modulating and processing the pain experience [63,64,65]. Similarly, the anterior insula exhibits both anatomical and functional connections with the limbic system and plays a role in affective processes [66]. Therefore, we believe that cortico-limbic system engagement also underlies the analgesic effects associated with VGAIT.

We also found altered thalamus rsFC with the occipital (visual cortex) and temporal lobes (auditory cortex), as well as motor areas (i.e., M1, SMA). As a gateway to the cerebral cortex, the thalamus is a relay station for the transmitting of multimodal sensory and motor information, including nociceptive information for pain consciousness [67]. 

The literature suggests that interactions of pain sensation with sensory input (e.g., vision, touch, and hearing) from the body offer the possibility to modulate chronic pain [68]. Several lines of study have revealed the effects of visual- and auditory-induced analgesia. Researchers found that visually induced illusory body distortions and images of arthritic body parts after being stretched or contracted have been shown to reduce chronic arthritic pain [69]. Similarly, visualizing images of acupuncture needle stimulation produced analgesia similar to that of real acupuncture manipulation [70]. Furthermore, sound-induced analgesia has been achieved in mice through a thalamus–auditory cortex pathway [71]. These modulations demonstrate that the sensory context of one’s own body alters the sensory processing and conscious experience of pain, thereby producing multisensory analgesia [72]. In accordance with these prior findings, our results suggest that VGAIT, a therapy based upon individuals imagining receiving acupuncture through visual stimuli, may act through multisensory and affective integration to induce pain relief.

The motor cortex (i.e., M1, SMA) is also implicated in the modulation of pain. Recent work suggests that M1 employs a layer-specific pathway through MD to attune sensory and aversive-emotional components of pain, which can be harnessed for pain relief [73]. More recently, motor cortex stimulation (MCS) has been applied for various pain syndromes such as chronic neuropathic pain, fibromyalgia, phantom limb pain, etc. [74,75,76]. The mechanism of MCS has been shown to modulate both descending and ascending pathways including thalamic areas [77]. Given this connection, the greater thalamus-motor cortex coupling induced by VGAIT may be related to pain-related adaptations in motor control.

### 4.3. Comparisons of Modulation Effects between Acupuncture and VGAIT on Thalamocortical Circuits

Both acupuncture and VGAIT elicited significant analgesic effects as indicated by pain threshold assessments. Notably, VGAIT demonstrated increased rsFC between the thalamus and key nodes within the interoceptive neural network and the task-positive network (TPN), which could be further divided into the salience network (i.e., ACC and insula) and central executive network (i.e., DLPFC, SMG, and PoCG), when compared to real acupuncture.

Pain is an embodied, personal, mental–emotional experience that has been referred to as an “embodied defense” [78]. The dysregulation of interoception, the basis of embodied processes, has been linked to chronic pain conditions [79,80]. The literature suggests that imagination can reactivate embodied pathways as part of the personal experience, and the thalamus may act as a bridge linking these forms of sensory perception [81]. From this perspective, VGAIT, a novel treatment involving imagining acupuncture sensations while watching a video of an acupuncturist manipulating needles on one’s body, can be regarded as an embodied acupuncture simulation meant to manipulate the body’s perception in an effort to reduce pain. 

The imagination process has been linked to the task-positive network (TPN), including the salience and central executive networks, which underlie focused attention and goal-directed activities [81,82]. We found increased rsFC between the thalamus and TPN in VGAIT compared to real acupuncture, suggesting that VGAIT is associated with a mechanism different from acupuncture. It is important to note that because VGAIT takes place in an embodied context compared to real acupuncture, engagement with interoceptive processing may promote additional physiological effects, as indicated by the activation of the TPN and interoceptive network. As such, thalamus–TPN coupling may serve as a possible neurological basis for VGAIT.

Studies have suggested that the TPN and DMN, two major brain networks, typically reveal anticorrelated connectivity in a resting state [82,83]. Interestingly, we found that real acupuncture enhanced rsFC between the thalamus and the DMN, whereas VGAIT increased rsFC between the thalamus and TPN (i.e., salience and executive networks). These findings suggest distinct thalamocortical pathways are associated with these two interventions.

Furthermore, we found that both acupuncture and VGAIT increased rsFC between the Mthal and the right parietal operculum, and temporal cortex, compared to sham acupuncture and VGAIT, respectively. The parietal operculum is involved in pain processing, attenuation, and the enhancement of ascending somatosensory information integration, which plays a crucial role in pain modulation [84]. The temporal lobe is involved in processing sensory input into derived meanings for the retention of visual memory, and emotion association [85]. A previous study suggested that the temporal association cortex is essential in integrating mental imagery-induced multisensory perception [86]. Our results implicate connectivity between the thalamus and parietal operculum/temporal cortex as a common neural mechanism underlying both acupuncture- and VGAIT-induced analgesia.

In addition, we found acupuncture and VGAIT increased rsFC between the MD and the right DLPFC, compared to pre-intervention. A recent study found that thalamus-DLPFC pathways might play a crucial role in regulating chronic pain and associated depression [87]. Thus, modulating rsFC between MD with the DLPFC may also represent a common mechanism underlying acupuncture and VGAIT in pain management. 

### 4.4. Limitations

The current study presents some limitations that should be addressed in future research. Firstly, the crossover design used in this study has the potential to influence results through participants’ awareness of the different treatment conditions. Future studies may consider applying a parallel design with a larger sample size, particularly for the treatment of chronic pain in the patient population. In addition, the study was conducted on healthy participants; it is therefore uncertain if the results and conclusions will be applicable to chronic pain populations. Further research is needed to examine the thalamocortical mechanisms underlying acupuncture and VGAIT in chronic pain patients.

## 5. Conclusions

We found that thalamocortical mechanisms are implicated in both acupuncture and VGAIT-induced analgesia. Since VGAIT can be applied remotely through online platforms, our results suggest that VGAIT may be a potential e-health treatment option for pain management.

## Figures and Tables

**Table 1:** Ventral posterolateral thalamus-based rsFC results across different interventions.

Contrasts (*n* = 24)		Brain Regions ^a^	Cluster Size	Peak z-Value	Peak MNI Coordinates		
					x	y	z
**Real-acu: post vs. pre**	***post > pre***	L Middle temporal gyrus	165	4.02	−68	−38	−6
		L Middle frontal gyrus	114	3.51	−38	14	56
	***pre > post***	*No regions survive the threshold*					
**Sham-acu: post vs. pre**	***post > pre***	*No regions survive the threshold*					
	***pre > post***	R Superior frontal gyrus	203	4.25	26	8	62
		R Calcarine	197	3.93	20	−104	−2
		R Postcentral gyrus	169	3.67	30	−42	46
**VGAIT: post vs. pre**	***post > pre***	L Postcentral gyrus	170	4.18	−54	−22	58
		L Superior parietal gyrus	151	3.58	−32	−44	68
	***pre > post***	*No regions survive the threshold*					
**VGAIT-control: post vs. pre**	***post > pre***	*No regions survive the threshold*					
	***pre > post***	R Postcentral gyrus	249	4.07	14	−52	68
		L Superior frontal gyrus	151	3.87	−24	0	66
**Real-acu vs. Sham-acu**		*No regions survive the threshold*					
**VGAIT vs. VGAIT control**	***post > pre***	L Postcentral gyrus	898	4.45	−32	−48	66
		R Postcentral gyrus	814	4.36	36	−38	50
		L Superior frontal gyrus	187	3.73	−24	−4	66
		R Superior frontal gyrus	136	4.04	20	−2	72
		R Superior frontal gyrus	107	4.31	28	−12	48
	***pre > post***	*No regions survive the threshold*					
**VGAIT vs. Real-acu**	***post > pre***	L Postcentral gyrus	369	4.04	−30	−42	68
		R Middle frontal gyrus	128	3.25	44	40	34
	***pre > post***	*No regions survive the threshold*					

**^a^** pairwise *t*-tests; results were significant at cluster level *p*_FDR_ < 0.05.

**Table 2:** Mediodorsal thalamus-based rsFC results across different interventions.

Contrasts (*n* = 24)		Brain Regions ^a^	Cluster Size	Peak z-Value	Peak MNI Coordinates		
					x	y	z
**Real-acu: post vs. pre**	***post > pre***	R Middle temporal gyrus	364	4.00	62	−30	−12
		L Angular gyrus	224	3.75	−50	−70	42
		R Angular gyrus	216	3.54	40	−62	42
		R Middle frontal gyrus	139	4.12	34	24	54
		R Inferior frontal gyrus	139	4.18	48	36	10
		R Cerebellar Lobule VI	98	3.66	8	−82	−16
		R Lingual gyrus	96	3.61	16	−74	−6
		L Middle occipital gyrus	87	4.47	−24	−86	6
		L Middle temporal gyrus	78	4.08	−66	−24	−8
	***pre > post***	L Precuneus	405	4.24	−12	−50	66
**Sham-acu: post vs. pre**	***post > pre***	*No regions survive the threshold*					
	***pre > post***	R Posterior cingulate cortex	389	4.13	6	−38	30
		Bil Cerebellum lobule IX	180	5.05	2	−46	−42
		L Middle temporal gyrus	121	3.63	−56	−18	−24
		R Middle temporal gyrus	111	3.64	68	−12	−12
**VGAIT: post vs. pre**	***post > pre***	R Anterior insula	388	3.97	38	4	2
		Bil Supplementary motor area	233	3.54	2	22	50
		R Superior temporal pole	208	4.42	46	20	−18
		L Anterior insula	192	4.05	−24	22	6
		L Anterior insula	153	4.06	−34	4	8
		L Middle frontal gyrus	143	3.83	−40	54	8
		R Middle frontal gyrus	114	3.45	42	36	14
		L Superior temporal gyrus	106	3.33	−52	−24	4
	***pre > post***	*No regions survive the threshold*					
**VGAIT control: post vs. pre**	***post > pre***	R Cerebellum lobule VI	181	4.44	30	−46	−28
	***pre > post***	R Superior temporal pole	152	4.85	54	16	−22
**Real-acu vs. Sham-acu**	***post > pre***	R Lingual gyrus	678	4.36	12	−100	−14
		R Middle temporal gyrus	510	4.62	66	−24	−12
		L Lingual gyrus	301	3.94	−26	−90	−20
		L Angular gyrus	200	4.01	−50	−70	42
		R Angular gyrus	193	4.43	40	−60	42
		R Middle frontal gyrus	121	4.14	38	14	62
		Bil Posterior cingulate gyrus	111	3.42	−2	−52	28
		L Parahippocampal gyrus	110	4.34	−34	−6	−26
	***pre > post***	L Precuneus	323	4.49	−10	−48	72
		R Middle cingulate gyrus	299	5.06	6	12	42
		R Middle frontal gyrus	104	3.95	34	50	30
		R Postcentral gyrus	103	3.57	20	−38	74
**VGAIT vs. VGAIT control**	***post > pre***	R Superior temporal pole	609	4.67	46	20	−20
		Bil Supplementary motor area/Anterior cingulate cortex	508	4.53	0	20	52
		R Middle frontal gyrus	244	3.99	44	50	14
		R Precentral gyrus	112	4.24	28	−10	50
		L Middle frontal gyrus	105	3.48	−38	52	6
	***pre > post***	*No regions survive the threshold*					
**VGAIT vs. Real-acu**	***post > pre***	Bil Anterior cingulate cortex	393	3.71	0	32	24
		R Anterior insula	293	4.25	38	6	2
		L Anterior insula	211	4.45	−36	4	6
		L Supramarginal gyrus	113	3.51	−56	−28	26
		L Middle frontal gyrus	105	3.57	−34	42	38
	***pre > post***	*No regions survive the threshold*					

**^a^** pairwise *t*-tests; results were significant at cluster level *p*_FDR_ < 0.05.

**Table 3:** Motor thalamus-based rsFC results across different interventions.

Contrasts (*n* = 24)		Brain Regions ^a^	Cluster Size	Peak z-Value	Peak MNI Coordinates		
					x	y	z
**Real-acu: post vs. pre**		*No regions survive the threshold*					
**Sham-acu: post vs. pre**	***post > pre***	*No regions survive the threshold*					
	***pre > post***	L Superior temporal gyrus	281	3.76	−68	−30	8
		R Superior temporal gyrus	231	4.16	44	−32	6
		R Rolandic operculum	172	4.03	52	−16	14
		R Middle temporal gyrus	153	4.98	48	−12	−18
		L Middle temporal gyrus	139	4.28	−60	2	−22
**VGAIT: post vs. pre**	***post > pre***	R Insula	1305	4.51	44	2	4
		R Middle cingulate cortex	881	4.55	6	14	36
		L Superior temporal gyrus	406	4.18	−62	−18	10
		R Supramarginal gyrus	233	4.12	50	−24	30
		L Insula	192	4.38	−30	2	12
		L Postcentral gyrus	146	3.80	−20	−26	72
		L Inferior parietal gyrus	121	3.62	−32	−54	40
	***pre > post***	*No regions survive the threshold*					
**VGAIT-control: post vs. pre**	***post > pre***	*No regions survive the threshold*					
	***pre > post***	R Superior temporal gyrus	149	4.12	44	−36	0
**Real-acu vs. Sham-acu**	***post > pre***	R Superior temporal gyrus	231	4.38	48	−30	8
		L Superior temporal gyrus	225	3.83	−50	−40	22
		L Middle temporal gyrus	135	3.86	−60	2	−24
		R Rolandic operculum	128	4.05	52	−16	16
		Bil Posterior cingulate cortex	114	4.06	0	−50	26
		R Medial prefrontal cortex	96	3.41	10	50	30
	***pre > post***	*No regions survive the threshold*					
**VGAIT vs. VGAIT control**	***post > pre***	R Postcentral gyrus	830	4.32	28	−10	52
		R Superior temporal gyrus	425	4.05	46	−36	−2
		L Superior temporal gyrus	354	4.72	−62	−18	10
		R Superior temporal gyrus	333	3.82	58	−4	4
		R Superior temporal pole	333	4.95	56	18	−8
		R Middle temporal gyrus	233	3.94	62	−58	10
		L Postcentral gyrus	146	3.68	−20	−26	72
		L Superior temporal gyrus	145	3.55	−62	−50	14
		L Precentral gyrus	140	4.27	−38	0	42
		R Precentral gyrus	132	4.01	58	8	38
		R Inferior temporal gyrus	114	4.00	50	−52	−14
		L Middle temporal gyrus	100	4.37	−38	−62	18
		R Inferior frontal gyrus	94	3.73	44	26	4
	***pre > post***	L Inferior occipital gyrus	140	4.01	−24	−84	−6
**VGAIT vs. Real-acu**	***post > pre***	R Insula	352	4.04	44	0	2
	***pre > post***	R Lingual gyrus	209	3.76	22	−76	2
		L Middle occipital gyrus	176	3.88	−26	−78	−2

**^a^** pairwise *t*-tests; results were significant at cluster level *p*_FDR_ < 0.05.

## Data Availability

The data that support the findings of this study are available from the corresponding author upon reasonable request.

## Supplementary Materials

The following supporting information can be downloaded at: https://www.mdpi.com/article/10.3390/biomedicines11071830/s1, detailed description of interventions and Table S1: Demographics and pain threshold assessments for all participants, [33,34,36,37,38,88,89].
