# Supplementary material for: Thalamocortical Mechanisms Underlying Real and Imagined Acupuncture
Source: Biomedicines. 2023 Jun 26;11(7):1830. doi: 10.3390/biomedicines11071830 (PMC10377130; doi:10.3390/biomedicines11071830)
Supplement: Supplementary file 1 [file biomedicines-11-01830-s001.zip › biomedicines-2455096-supplementary.pdf]

Supplementary Material

(Thalamocortical Mechanisms Underlying Real and Imagined Acupuncture)

1. Detailed description of interventions

Real acupuncture procedure

Real acupuncture was applied on the right acupoints SP6 (Sanyinjiao) and SP9 (Yinlingquan) in a standardized manner, with parameters of needling were kept consistent for all participants: 1-2 cm needling depth, 120 rotations / minute, and a 90° insertion angle. Each acupoint was given 10 second rotations followed by a 30-second break.

Sham acupuncture procedure

Sham acupuncture was applied at two sham points (adjacent to SP6/SP9) using a specially designed needle that did not penetrate the skin (Streitberger needle). This placebo needle has been previously demonstrated to be undistinguishable from genuine needling by participants [1,2]. These two sham points were located on the leg where no meridian passed through (i.e., posterior and superior 1/3 of K9 and K10, and 1 cun posterior to K8). The sham acupuncture intervention was applied using the same paradigm as the real acupuncture.

Video-guided acupuncture imagery treatment (VGAIT)

At the beginning of the VGAIT session, participants were introduced to imagery acupuncture intervention outside of the MRI scanner. They were given instructions to read which stated: "You will see a video of acupuncture intervention being applied on your leg. Please focus on the needle manipulation and try to imagine here is an actual needle being placed into your leg at the same spot. You will find that you can actually feel the needle manipulation on your leg at the same spot as in the video, as well as some soreness and an aching, dull pain along with other sensations. It is very important that you stay focused and try to imagine the sensation of receiving acupuncture as vividly as possible." Following these instructions, the VGAIT procedure was then applied during the fMRI scan.

VGAIT control condition

The VGAIT control condition followed the same protocol as VGAIT, with the exception that cotton swabs were used to repeatedly touch non-acupoints, and these swabs were gently rotated following the same procedure as in the real and sham acupuncture conditions. Participants were provided with instructions beforehand, which stated that: "You will see a video of a swab touching your leg. Please focus on the cotton swab and try to imagine there is an actual swab being placed on your leg at the same spot. You will find that you can actually feel the cotton swab on your leg at the same spot as in the video. It is very important that you stay focused and try to imagine the sensation of the swab as vividly as possible." Upon reading such instructions, the VGAIT control procedure was performed during the fMRI scan.

2. Pain threshold measurements

The heat and pressure pain thresholds were assessed before and after each intervention in a

quiet room outside of the scanner. We chose two pain modalities because heat-evoked pain is predominantly mediated by small, nonmyelinated peripheral nociceptive nerve fibers (C-fibers), whereas pressure-evoked pain is predominantly mediated by small, myelinated peripheral nociceptive nerve fibers (A-delta fibers) [3,4]. Pain threshold assessments were applied at two locations (leg and arm / thumbnail), each measured three times, with the thermode (heat) and algometer (pressure) repositioned between each threshold assessments. Heat-evoked pain thresholds were assessed using a PATHWAY CHEPS (Contact Heat-Evoked Potential Stimulator, Medoc Advanced Medical Systems) [5], and pressure-evoked pain thresholds were assessed using an algometer [6]. A detailed description of the Quantitative sensory test (QST) procedure can be found in our previous publication [7].

3. Detailed Quantitative Sensory Testing (QST) results

Supplementary Table 1. Demographics and pain threshold assessments for all participants

	Real acupuncture	Sham acupuncture	VGAIT	VGAIT control
Demographics				
N	24	—	—	—
Age (years)	25.21 ± 3.83	—	—	—
Sex (Female)	16	—	—	—
Quantitative sensory test (QST)				
Heat pain threshold on leg				
Pre- intervention	42.76 ± 3.47	42.49 ± 2.95	42.44 ± 3.55	42.04 ± 3.27
Post- intervention	43.73 ± 3.45	42.39 ± 3.07	43.31 ± 3.77	42.26 ± 3.63
Difference (post minus pre)	0.97 ± 1.74	-0.10 ± 1.41	0.87 ± 1.66	0.21 ± 1.56
Heat pain threshold on arm				
Pre- intervention	41.04 ± 3.15	40.61 ± 2.78	41.31 ± 3.38	41.01 ± 3.29
Post- intervention	42.25 ± 2.83	40.79 ± 3.00	41.88 ± 3.34	41.20 ± 3.17
Difference (post minus pre)	1.21 ± 1.19	0.18 ± 1.40	0.57 ± 1.62	0.19 ± 0.83
Pressure pain threshold on leg				
Pre- intervention	6.10 ± 2.21	5.56 ± 2.01	5.38 ± 1.78	5.63 ± 1.79
Post- intervention	6.84 ± 1.84	5.29 ± 1.83	6.38 ± 1.93	5.26 ± 1.85
Difference (post minus pre)	0.74 ± 1.33	-0.28 ± 0.91	1.00 ± 0.99	-0.36 ± 0.51
Pressure pain threshold on thumbnail				
Pre- intervention	5.99 ± 1.83	5.65 ± 1.98	5.49 ± 1.60	5.87 ± 1.55
Post- intervention	7.13 ± 1.79	5.43 ± 1.60	6.61 ± 1.65	5.81 ± 1.76
Difference (post minus pre)	1.14 ± 0.82	-0.22 ± 0.89	1.12 ± 1.10	-0.07 ± 0.67

Notes: Pain thresholds for the four interventions are shown with the mean ± standard deviation (SD). Abbreviations: VGAIT, video-guided acupuncture imagery treatment.

References

1. Kong, J.; Gollub, R.L.; Rosman, I.S.; Megan Webb, J.; Vangel, M.G.; Kirsch, I.; Kaptchuk, T.J. Brain Activity Associated with Expectancy-Enhanced Placebo Analgesia as Measured by Functional Magnetic Resonance Imaging. *J Neurosci* **2006**, *26*, 381–388, doi:10.1523/JNEUROSCI.3556-05.2006.
2. Streitberger, K.; Kleinhenz, J. Introducing a Placebo Needle into Acupuncture Research. *Lancet* **1998**, *352*, 364–365, doi:10.1016/S0140-6736(97)10471-8.
3. Angst, M.S.; Tingle, M.; Phillips, N.G.; Carvalho, B. Determining Heat and Mechanical Pain Threshold in Inflamed Skin of Human Subjects. *J Vis Exp* **2009**, doi:10.3791/1092.
4. Beissner, F.; Brandau, A.; Henke, C.; Felden, L.; Baumgärtner, U.; Treede, R.D.; Oertel, B.G.; Lötsch, J. Quick Discrimination of A(Delta) and C Fiber Mediated Pain Based on Three Verbal Descriptors. *PLoS One* **2010**, *5*, doi:10.1371/JOURNAL.PONE.0012944.
5. Kong, J.; Spaeth, R.; Cook, A.; Kirsch, I.; Claggett, B.; Vangel, M.; Gollub, R.L.; Smoller, J.W.; Kaptchuk, T.J. Are All Placebo Effects Equal? Placebo Pills, Sham Acupuncture, Cue Conditioning and Their Association. *PLoS One* **2013**, *8*, doi:10.1371/JOURNAL.PONE.0067485.
6. Schabrun, S.M.; Jones, E.; Elgueta Cancino, E.L.; Hodges, P.W. Targeting Chronic Recurrent Low Back Pain from the Top-down and the Bottom-up: A Combined Transcranial Direct Current Stimulation and Peripheral Electrical Stimulation Intervention. *Brain Stimul* **2014**, *7*, 451–459, doi:10.1016/J.BRS.2014.01.058.
7. Tu, Y.; Zhang, B.; Cao, J.; Wilson, G.; Zhang, Z.; Kong, J. Identifying Inter-Individual Differences in Pain Threshold Using Brain Connectome: A Test-Retest Reproducible Study. *Neuroimage* **2019**, *202*, doi:10.1016/J.NEUROIMAGE.2019.116049.
